# Correction: *ai-corona*: Radiologist-assistant deep learning framework for COVID-19 diagnosis in chest CT scans

**DOI:** 10.1371/journal.pone.0257119

**Published:** 2021-09-01

**Authors:** Mehdi Yousefzadeh, Parsa Esfahanian, Seyed Mohammad Sadegh Movahed, Saeid Gorgin, Dara Rahmati, Atefeh Abedini, Seyed Alireza Nadji, Sara Haseli, Mehrdad Bakhshayesh Karam, Arda Kiani, Meisam Hoseinyazdi, Jafar Roshandel, Reza Lashgari

One of the assigned affiliations for the fifth author is incorrect. Dara Rahmati is not affiliated with #1, but with #5: Department of Computer Science and Engineering, Shahid Beheshti University, Tehran, Iran.
