# Supplementary figures and images for: ai-corona: Radiologist-assistant deep learning framework for COVID-19 diagnosis in chest CT scans
Source: PLoS One. 2021 May 7;16(5):e0250952. doi: 10.1371/journal.pone.0250952 (PMC8104381; doi:10.1371/journal.pone.0250952)

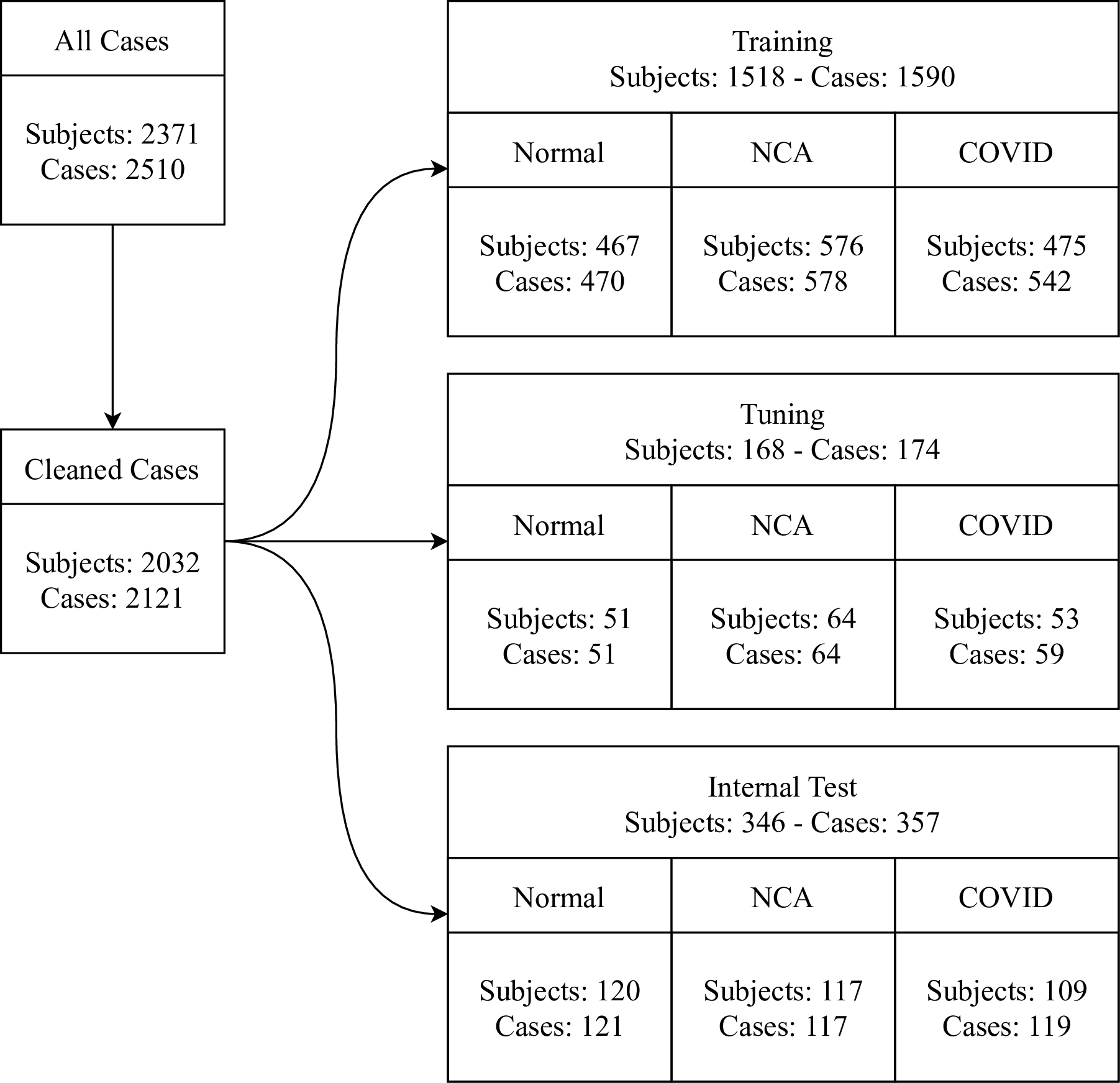

Supplement: S1 Fig — The preliminary dataset was cleaned, by removing abdomen and high-resolution CT scans. The train and tuning sets were labeled by two expert radiologists. The NCA and Normal classes of the test set was re-annotated by three expert radiologist (one new). The COVID-19 class are patients that meet our criteria and were hospitalized for more than three days. (TIF) [file pone.0250952.s001.tif]

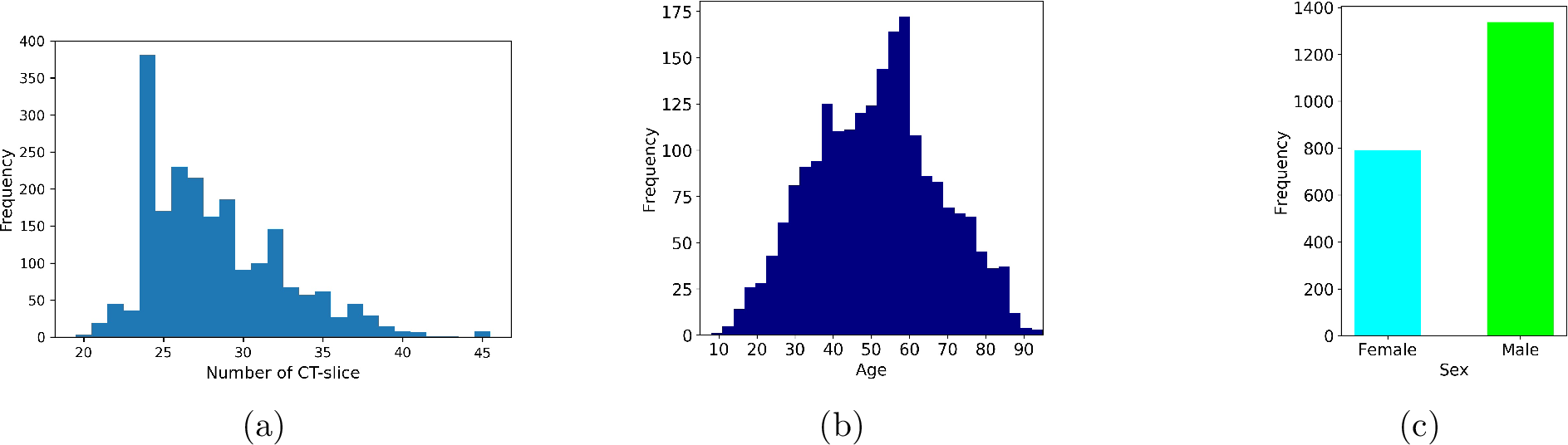

Supplement: S2 Fig — (TIF) [file pone.0250952.s002.tif]

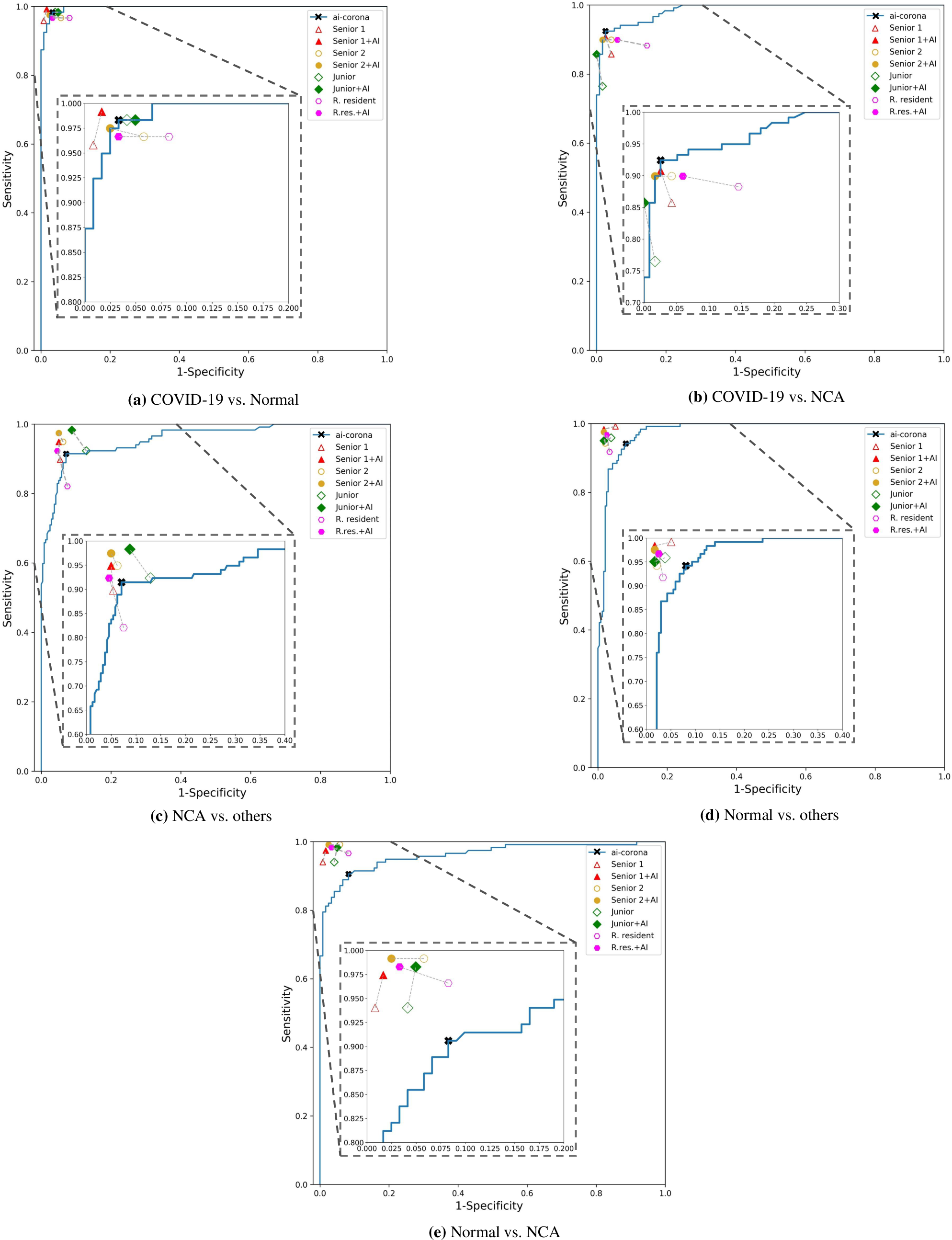

Supplement: S3 Fig — The Solid black line is for ai-corona by adapting different discrimination threshold value which is used to convert the continuous probability to binary “Yes” or “No” results. The filled triangle symbols are the (1-specificity, sensitivity) for the individual clinical experts, while the filled circle symbols are for the model-assisted radiologist. The inset plots magnify the highest part of sensitivity and specificity. (TIF) [file pone.0250952.s003.tif]
