# Supplementary material for: ai-corona: Radiologist-assistant deep learning framework for COVID-19 diagnosis in chest CT scans
Source: PLoS One. 2021 May 7;16(5):e0250952. doi: 10.1371/journal.pone.0250952 (PMC8104381; doi:10.1371/journal.pone.0250952)
Supplement: S1 Table — (PDF) [file pone.0250952.s004.pdf]

S1 Table. The quantitative evaluation of *ai-corona*, radiologists, and AI-assisted radiologists' performance results for differentiating between the COVID-19 class and the Normal class at a 95% confidence interval.

	Sensitivity (95% CI)	Specificity (95% CI)	F1-score (95% CI)	Kappa (95% CI)
ai-corona	0.983 (0.971, 0.995)	0.967 (0.951, 0.983)	0.975 (0.965, 0.985)	0.950 (0.929, 0.971)
Senior 1	0.958 (0.947, 0.969)	0.992 (0.987, 0.997)	0.974 (0.967, 0.981)	0.950 (0.938, 0.962)
Senior 1 + AI	0.992 (0.987, 0.997)	0.983 (0.976, 0.990)	0.987 (0.983, 0.991)	0.975 (0.966, 0.984)
Senior 2	0.966 (0.957, 0.975)	0.942 (0.930, 0.954)	0.954 (0.944, 0.964)	0.908 (0.892, 0.924)
Senior 2 + AI	0.975 (0.967, 0.983)	0.975 (0.966, 0.984)	0.975 (0.969, 0.981)	0.950 (0.938, 0.962)
Junior	0.983 (0.977, 0.989)	0.959 (0.949, 0.969)	0.971 (0.965, 0.977)	0.942 (0.930, 0.954)
Junior + AI	0.983 (0.976, 0.990)	0.950 (0.939, 0.961)	0.967 (0.960, 0.974)	0.933 (0.919, 0.947)
R. Resident	0.966 (0.957, 0.975)	0.917 (0.904, 0.93)	0.943 (0.934, 0.952)	0.883 (0.867, 0.899)
R. Res. + AI	0.966 (0.957, 0.975)	0.967 (0.956, 0.978)	0.966 (0.959, 0.973)	0.933 (0.919, 0.947)
