# Supplementary material for: ai-corona: Radiologist-assistant deep learning framework for COVID-19 diagnosis in chest CT scans
Source: PLoS One. 2021 May 7;16(5):e0250952. doi: 10.1371/journal.pone.0250952 (PMC8104381; doi:10.1371/journal.pone.0250952)
Supplement: S2 Table — (PDF) [file pone.0250952.s005.pdf]

S2 Table. The quantitative evaluation of *ai-corona*, radiologists, and AI-assisted radiologists' performance results for differentiating between the COVID-19 class and the NCA class at a 95% confidence interval.

	Sensitivity (95% CI)	Specificity (95% CI)	F1-score (95% CI)	Kappa (95% CI)
ai-corona	0.924 (0.901, 0.947)	0.974 (0.959, 0.989)	0.949 (0.934, 0.964)	0.898 (0.872, 0.924)
Senior 1	0.857 (0.836, 0.878)	0.957 (0.944, 0.970)	0.903 (0.890, 0.916)	0.814 (0.794, 0.834)
Senior 1 + AI	0.908 (0.892, 0.924)	0.974 (0.965, 0.983)	0.939 (0.929, 0.949)	0.881 (0.865, 0.897)
Senior 2	0.899 (0.884, 0.914)	0.957 (0.946, 0.968)	0.926 (0.916, 0.936)	0.856 (0.837, 0.875)
Senior 2 + AI	0.899 (0.881, 0.917)	0.983 (0.976, 0.990)	0.939 (0.929, 0.949)	0.881 (0.863, 0.899)
Junior	0.765 (0.743, 0.787)	0.983 (0.974, 0.992)	0.858 (0.843, 0.873)	0.746 (0.723, 0.769)
Junior + AI	0.857 (0.839, 0.875)	1.000 (1.000, 1.000)	0.923 (0.912, 0.934)	0.856 (0.837, 0.875)
R. Resident	0.882 (0.863, 0.901)	0.855 (0.838, 0.872)	0.871 (0.858, 0.884)	0.737 (0.711, 0.763)
R. Res. + AI	0.899 (0.882, 0.916)	0.940 (0.927, 0.953)	0.918 (0.906, 0.930)	0.839 (0.815, 0.863)
