# Supplementary material for: ai-corona: Radiologist-assistant deep learning framework for COVID-19 diagnosis in chest CT scans
Source: PLoS One. 2021 May 7;16(5):e0250952. doi: 10.1371/journal.pone.0250952 (PMC8104381; doi:10.1371/journal.pone.0250952)
Supplement: S3 Table — (PDF) [file pone.0250952.s006.pdf]

S3 Table. The quantitative evaluation of *ai-corona*, radiologists, and AI-assisted radiologists' performance results for differentiating between the NCA class and the other classes at a 95% confidence interval.

	Sensitivity (95% CI)	Specificity (95% CI)	F1-score (95% CI)	Kappa (95% CI)
ai-corona	0.915 (0.883, 0.947)	0.929 (0.893, 0.965)	0.922 (0.894, 0.950)	0.831 (0.793, 0.869)
Senior 1	0.897 (0.876, 0.918)	0.946 (0.925, 0.967)	0.894 (0.877, 0.911)	0.841 (0.815, 0.867)
Senior 1 + AI	0.949 (0.934, 0.964)	0.950 (0.930, 0.970)	0.925 (0.911, 0.939)	0.887 (0.870, 0.904)
Senior 2	0.949 (0.934, 0.964)	0.938 (0.916, 0.960)	0.914 (0.900, 0.928)	0.869 (0.848, 0.890)
Senior 2 + AI	0.974 (0.963, 0.985)	0.950 (0.932, 0.968)	0.938 (0.926, 0.950)	0.906 (0.890, 0.922)
Junior	0.923 (0.901, 0.945)	0.871 (0.843, 0.899)	0.844 (0.824, 0.864)	0.757 (0.733, 0.781)
Junior + AI	0.983 (0.974, 0.992)	0.912 (0.890, 0.934)	0.909 (0.896, 0.922)	0.860 (0.838, 0.882)
R. Resident	0.821 (0.793, 0.849)	0.925 (0.897, 0.953)	0.831 (0.810, 0.852)	0.750 (0.723, 0.777)
R. Res. + AI	0.923 (0.904, 0.942)	0.954 (0.935, 0.973)	0.915 (0.901, 0.929)	0.873 (0.854, 0.892)
