# Supplementary material for: ai-corona: Radiologist-assistant deep learning framework for COVID-19 diagnosis in chest CT scans
Source: PLoS One. 2021 May 7;16(5):e0250952. doi: 10.1371/journal.pone.0250952 (PMC8104381; doi:10.1371/journal.pone.0250952)
Supplement: S4 Table — (PDF) [file pone.0250952.s007.pdf]

S4 Table. The quantitative evaluation of *ai-corona*, radiologists, and AI-assisted radiologists' performance results for differentiating between the Normal class and the other classes at a 95% confidence interval.

	Sensitivity (95% CI)	Specificity (95% CI)	F1-score (95% CI)	Kappa (95% CI)
ai-corona	0.942 (0.917, 0.967)	0.919 (0.883, 0.955)	0.931 (0.907, 0.955)	0.841 (0.801, 0.881)
Senior 1	0.992 (0.985, 0.999)	0.949 (0.929, 0.969)	0.949 (0.937, 0.961)	0.920 (0.904, 0.936)
Senior 1 + AI	0.983 (0.975, 0.991)	0.983 (0.971, 0.995)	0.975 (0.967, 0.983)	0.963 (0.952, 0.974)
Senior 2	0.942 (0.926, 0.958)	0.979 (0.964, 0.994)	0.950 (0.941, 0.959)	0.925 (0.909, 0.941)
Senior 2 + AI	0.975 (0.964, 0.986)	0.983 (0.971, 0.995)	0.971 (0.962, 0.980)	0.956 (0.944, 0.968)
Junior	0.959 (0.946, 0.972)	0.962 (0.943, 0.981)	0.943 (0.931, 0.955)	0.913 (0.896, 0.930)
Junior + AI	0.950 (0.933, 0.967)	0.983 (0.970, 0.996)	0.958 (0.947, 0.969)	0.937 (0.921, 0.953)
R. Resident	0.917 (0.899, 0.935)	0.966 (0.949, 0.983)	0.925 (0.911, 0.939)	0.887 (0.866, 0.908)
R. Res. + AI	0.967 (0.954, 0.980)	0.975 (0.962, 0.988)	0.959 (0.950, 0.968)	0.938 (0.923, 0.953)
