# Supplementary material for: ai-corona: Radiologist-assistant deep learning framework for COVID-19 diagnosis in chest CT scans
Source: PLoS One. 2021 May 7;16(5):e0250952. doi: 10.1371/journal.pone.0250952 (PMC8104381; doi:10.1371/journal.pone.0250952)
Supplement: S5 Table — (PDF) [file pone.0250952.s008.pdf]

S5 Table. The quantitative evaluation of *ai-corona*, radiologists, and AI-assisted radiologists' performance results for differentiating between the NCA class and the Normal class at a 95% confidence interval.

	Sensitivity (95% CI)	Specificity (95% CI)	F1-score (95% CI)	Kappa (95% CI)
ai-corona	0.906 (0.878, 0.934)	0.917 (0.891, 0.943)	0.912 (0.893, 0.931)	0.823 (0.789, 0.857)
Senior 1	0.940 (0.927, 0.953)	0.992 (0.986, 0.998)	0.965 (0.958, 0.972)	0.933 (0.921, 0.945)
Senior 1 + AI	0.974 (0.966, 0.982)	0.983 (0.976, 0.990)	0.979 (0.973, 0.985)	0.958 (0.948, 0.968)
Senior 2	0.991 (0.987, 0.995)	0.942 (0.930, 0.954)	0.967 (0.960, 0.974)	0.933 (0.920, 0.946)
Senior 2 + AI	0.991 (0.986, 0.996)	0.975 (0.966, 0.984)	0.983 (0.978, 0.988)	0.966 (0.957, 0.975)
Junior	0.940 (0.928, 0.952)	0.959 (0.949, 0.969)	0.948 (0.938, 0.958)	0.899 (0.879, 0.919)
Junior + AI	0.983 (0.976, 0.990)	0.950 (0.939, 0.961)	0.966 (0.960, 0.972)	0.933 (0.919, 0.947)
R. Resident	0.966 (0.957, 0.975)	0.917 (0.903, 0.931)	0.942 (0.933, 0.951)	0.882 (0.866, 0.898)
R. Res. + AI	0.983 (0.977, 0.989)	0.967 (0.957, 0.977)	0.975 (0.969, 0.981)	0.950 (0.940, 0.960)
