# Supplementary material for: ai-corona: Radiologist-assistant deep learning framework for COVID-19 diagnosis in chest CT scans
Source: PLoS One. 2021 May 7;16(5):e0250952. doi: 10.1371/journal.pone.0250952 (PMC8104381; doi:10.1371/journal.pone.0250952)
Supplement: S6 Table — (PDF) [file pone.0250952.s009.pdf]

S6 Table. *ai-corona*'s pairwise and multi-class diagnosis AUC for the MDH cohort.

	AUC
NCA vs others	0.959 (0.944, 0.974)
Normal vs others	0.978 (0.968, 0.988)
COVID-19 vs Normal	0.997 (0.995, 0.999)
COVID-19 vs NCA	0.986 (0.981, 0.991)
Normal vs NCA	0.961 (0.951, 0.971)
