# Supplementary material for: ai-corona: Radiologist-assistant deep learning framework for COVID-19 diagnosis in chest CT scans
Source: PLoS One. 2021 May 7;16(5):e0250952. doi: 10.1371/journal.pone.0250952 (PMC8104381; doi:10.1371/journal.pone.0250952)
Supplement: Attachment — Submitted filename: PONE-D-21-04656.docx [file pone.0250952.s010.docx]

Manuscript number: **PONE-D-21-04656**

Article Title: ai-corona: Deep Radiologist-Assistant for COVID-19 Diagnosis in Chest CT Scans

**COMMENTS FOR AUTHORS**

**Minor Revision**

\# In the introductory section, the first paragraph should discuss about COVID-19 pandemic, number cases, deaths, recovered and the taxonomy of the virus etc.

\# Author should stress on why their contribution stand out or achieved better result than others.

\# There should be bullet points on authors contribution at the end of introduction.

\# Author should add a paragraph on COVID-19 vaccines.

\# Author need to add a section on “Related work” and a table to summarized contributions in terms of dataset and performance evaluation.

\# Author need to add a limitations and future work.

\# Authors can also add these references

1. Chowdhury, M. E., Rahman, T., Khandakar, A., Mazhar, R., Kadir, M. A., Mahbub, Z. B., ... & Islam, M. T. (2020). Can AI help in screening viral and COVID-19 pneumonia?. IEEE Access, 8, 132665-132676.
2. Ibrahim, A. U., Ozsoz, M., Serte, S., Al-Turjman, F., & Yakoi, P. S. (2021). Pneumonia classification using deep learning from chest X-ray images during COVID-19. Cognitive Computation, 1-13.
3. Burki, T. K. (2020). The Russian vaccine for COVID-19. The Lancet Respiratory Medicine, 8(11), e85-e86.
